# Pneumonia hospitalisations in Scotland following the introduction of pneumococcal conjugate vaccination in young children

**DOI:** 10.1186/s12879-016-1693-x

**Published:** 2016-08-09

**Authors:** Harish Nair, Arun Thor Watts, Linda J. Williams, Saad B. Omer, Colin R. Simpson, Lorna J. Willocks, J. Claire Cameron, Harry Campbell

**Affiliations:** 1 Centre for Global Health Research, Usher Institute of Population Health Sciences and Informatics, Edinburgh Medical School, University of Edinburgh, Teviot Place, Edinburgh, EH8 9AG UK; 2 Centre for Population Health Sciences, Usher Institute of Population Health Sciences and Informatics, Edinburgh Medical School, University of Edinburgh, Edinburgh, UK; 3 Hubert Department of Global Health, Rollins School of Public Health, Emory University, Atlanta, USA; 4 Centre for Medical Informatics, Usher Institute of Population Health Sciences and Informatics, Edinburgh Medical School, University of Edinburgh, Edinburgh, UK; 5 Directorate for Public Health and Health Policy, NHS Lothian, Edinburgh, UK; 6 Health Protection Scotland, NHS National Services Scotland, Glasgow, UK; 7 Public Health Foundation of India, New Delhi, India

**Keywords:** Pneumococcal conjugate vaccine, Indirect effects, All-cause pneumonia hospitalisations, Pneumococcal hospitalisations, Pneumonia mortality

## Abstract

**Background:**

Scotland introduced PCV7 and PCV13 immunisation in young children in 2006 and 2010 respectively. One recent study from the United States reported a decrease in hospitalisation rates for all-cause pneumonia most notably in adults older than 75 years of age following PCV7 introduction in the US child population. We aimed to examine the effect of PCV7 and PCV13 on hospitalisation rates for all-cause pneumonia across all age groups in Scotland.

**Methods:**

We linked hospital records and death certification datasets for the entire Scottish population for the period 2000 to 2012. We included all cases where the primary / secondary diagnosis was pneumonia. Differences in hospital admission rates for pneumonia by age group were calculated using the difference in average annual rates for each period.

**Results:**

We estimated that all-cause pneumonia hospitalisation rates in children <2 years decreased by about 30 % in the post-PCV-13 period compared with the pre-PCV period. However, in adults aged 75–84 years and ≥85 years, all-cause pneumonia hospitalisation rates increased by 63 and 46 % respectively in the post-PCV 13 period compared to the pre-PCV period. This resulted in an additional 7000 hospitalisations across all age groups in Scotland in 2012 about half of which were in adults >75 years. At the same time, the median length of hospital stay decreased by a third in children <2 years and by about 20 % in adults >75 years in the post-PCV13 period compared to the pre-PCV period. Additionally, there was an 11 % reduction in deaths due to all-cause pneumonia, and 30 % reduction in pneumococcal hospitalisations across all age groups in the post-PCV13 period compared with pre-PCV period.

**Discussion:**

The modest and sustained decline in the rates of hospitalisation for all-cause pneumonia in children and the reduction in proportion of pneumonia hospitalisations in children coded as pneumococcal disease in the post-PCV period should alleviate concerns that pneumococcal serotype replacement may have resulted in an increased pneumonia burden in this age group. The indirect impact of child PCV immunisation in those not vaccinated (in terms of reduction in all-cause pneumonia hospitalisations in the elderly) has not been seen in Scotland. Our results are likely to be confounded by changes in clinical coding and healthcare practices over the same period.

**Conclusions:**

Our results illustrate that health care planners cannot, with confidence, predict indirect PCV vaccine impacts on hospitalisations. IPD surveillance across all age groups is needed to assess the indirect effects of PCV in the community.

**Electronic supplementary material:**

The online version of this article (doi:10.1186/s12879-016-1693-x) contains supplementary material, which is available to authorized users.

## Background

Globally, lower respiratory tract infections (primarily pneumonia) were the second leading cause of morbidity and fourth leading cause of mortality across all age groups in 2013 [1]. The burden of pneumonia is disproportionately higher in young children and the elderly (above 65 years of age) [2, 3]. In general, mortality due to pneumonia across all ages has declined substantially (22 (95 % CI 16 to 30) percent) between 1990 and 2013 [1]. Prior to the introduction of the pneumococcal conjugate vaccine (PCV) in the US, hospitalisation rates for pneumonia in the elderly (65 to 84 years) increased by about 20 % in the 15 year period between 1988–1990 and 2000–2002 [3]. Similar increases (over a similar though abbreviated period) have been reported in England, Denmark, and the Netherlands [4–6]. The increase in this age group has been attributed to an ageing population, increase in the prevalence of comorbidities, and changes in coding and hospital admission practices [4].

The prevalence of *Streptococcus pneumoniae* in adults with community-acquired pneumonia (CAP) has declined sharply since the advent of antibiotics. Data from Medicare (for year 2009) indicate that about 7.6 % of CAP hospitalisations in the elderly (aged 65 years and above) in the United States can be attributed to *Streptococcus pneumoniae* [7]. Therefore, pneumococcal polysaccharide vaccine (PPV) was introduced in Scotland in the winter of 2003 to persons aged 65 years and above, and high risk individuals (i.e., those with co-morbidities). Following the introduction of PCV7 into the national child immunisation schedules in the US and England and Wales, there has been a decrease in the overall incidence of invasive pneumococcal disease (IPD) across all age groups [8, 9]. PCV7 was introduced into the Scottish child immunisation schedule in September 2006 at the age of 2 and 4 months with a booster dose at around 13 months. Severe pneumococcal disease (using IPD as a surrogate) was increasing in Scotland in the period prior to introduction of PCV7 (from 559 cases in 2000 to 753 cases in 2006) [10]. After the introduction of PCV7, and PCV13 later in 2010 (both using 2 + 1 dose schedule), there has been a decrease in the number of IPD cases in children younger than 5 years (from 753 cases in 2006 to 482 cases in 2012). It was expected that with the increased uptake of PCV, there would be an increase in the circulating non-vaccine serotypes (NVT) [11, 12]. In the US, after the introduction of PCV7, there was an increase in disease (across all age groups especially in young children and elderly) caused by NVT, most notably serotype 19 A [13]. In addition, in 2013, Griffin and colleagues demonstrated a decrease in hospitalisation rates for all-cause pneumonia (henceforth referred to as pneumonia) not only in children but also across all age groups, most notably in adults older than 75 years of age following introduction PCV7 in the US child population [14]. We aimed to examine the effect of PCV7 and PCV13 on hospitalisation rates for pneumonia across all age groups in Scotland and examine the generalizability of Griffin and colleagues’ findings.

## Methods

### Data sources

Scotland has an excellent health service data system with an ability to perform high quality linkage (through a unique personal identifier attached to health records – the Community Health Index [CHI] number) of individual hospitalisation, laboratory and death certification data which permits individual patient data analysis and follow-up. National Services Scotland is responsible for maintaining these data and ensuring high data quality, consistency and coverage and appropriate levels of data governance (http://www.isdscotland.org/). The Scottish Morbidity Record (SMR01) comprises episode level data on hospital inpatient and day case discharges from acute specialties from hospitals in Scotland. The dataset contains patient identifiers such as the CHI number, demographic details such as postcode together with health record data (episode management details and general clinical information). These data have been available electronically (for research purposes) since 1981. Usually, when inpatients and day cases are transferred to another specialty within the same hospital or are transferred to another hospital, a new record on SMR01 is generated. However, in this analysis, we used continuous inpatient stay (CIS) data by linking SMR01 episodes for each patient. This created “linked” individual patient histories (which includes transfers between hospitals, specialties, and consultants). This integrated measure documents the length of continuous hospital care episodes. Data from SMR01 on hospital admissions for pneumonia, hospitalisation, discharge diagnosis of pneumococcal pneumonia, readmissions within 14 days, sex, age, health board area, length of stay; and mortality data from General Register Office of Scotland (GRO99B) were collected.

### Definition of hospitalisation for pneumonia

Hospitalisation for pneumonia was defined in accordance with ICD 10 procedures. CIS data were selected for inclusion in the study if the patient had a primary diagnosis of pneumonia OR a primary diagnosis of sepsis, meningitis or empyema, with a diagnosis of pneumonia in any subsequent diagnostic position. A list of ICD 10 codes used to qualify diagnosis for all-cause pneumonia, meningitis, empyema, septicemia and pneumococcal pneumonia are detailed in Additional file 1: Table S1. We considered hospital readmissions within 14 days of the primary admission as part of the same event [15].

### Statistical analysis

SMR01 data from 2000 through 2012 (epidemiological year July-June) were used as numerator data in estimating annual hospitalisation rates for pneumonia. We constructed a cohort of patients by linking hospital records and death certification datasets. We used the following age groups for analysis: children aged below two years, 2 to 4 years, 5 to 17 years, adults aged 18 to 39 years, 40 to 64 years, 65 to 74 years, 75 to 84 years, and 85 years or older. We stratified the analysis into three different time periods: 2000 through 2005 were considered as the pre-PCV period; 2006 was treated as transition year, as this was the year that PCV was introduced nationally and therefore excluded from the analysis; 2007 through 2009 as the post-PCV7 period; and 2010 through 2012 as the post-PCV13 period (as PCV 13 was introduced nationally in April 2010). We used population by Scottish NHS Health Board of residence provided by ISD (based on official national census data and estimates) as population denominator data in the calculation of annual hospital admission rates (by age group) and reported these per 100,000 persons per year. Differences in hospital admission rates by age group were calculated using the average annual rates for each period, and the observed and expected rates were compared. We used these data to derive the percentage difference in relative rates. Where no data were available for a particular health board (age/sex/year combination), most frequently seen in the case of the smallest Health Boards, we assumed that there were no cases of pneumonia, rather than regarding these as missing data. Where the Health Board was given as “Other”, we considered that these could be due to two reasons: data were missing but the patient was resident in Scotland; or patient was a resident of another country but was treated in Scotland. We considered both scenarios in the analysis.

All data analyses were conducted using SPSS V19.0 (IBM, NY, USA) and SAS V9.2 (SAS institute Inc., NC, USA).

## Results

**Fig. 1:**
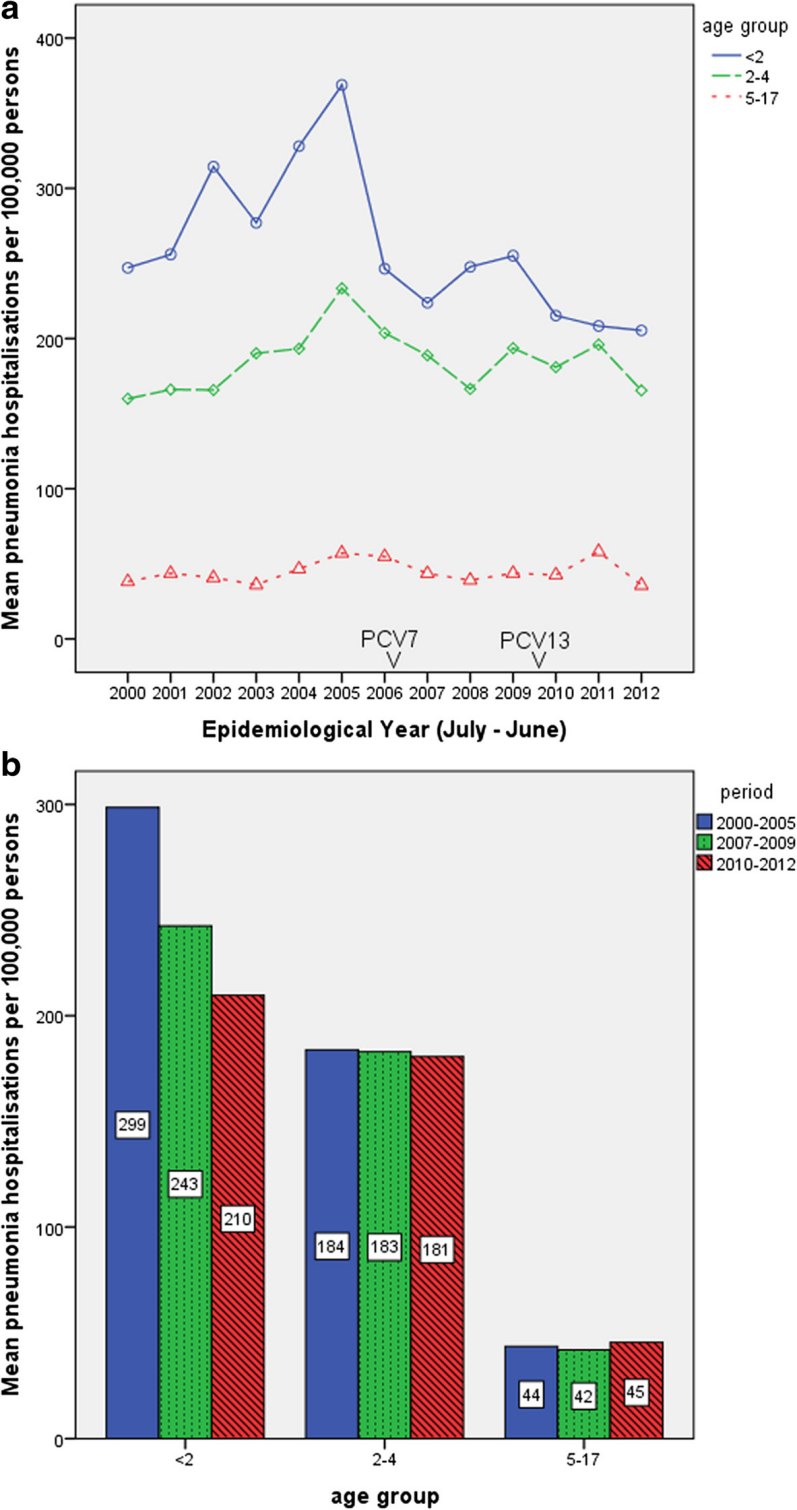
Pneumonia hospitalisations in Scotland from July 2000 to June 2012 in children. Panel **a** shows annual rates of hospitalisation for pneumonia from 2000 to 2012. PCV 7 was introduced in 2006 and PCV 13 in 2010. Panel **b** shows annual rates of hospitalisation for 2000 to 2005, 2007 to 2009 and 2010 to 2012 for all children (including those coded as “other” health boards)

**Fig. 2:**
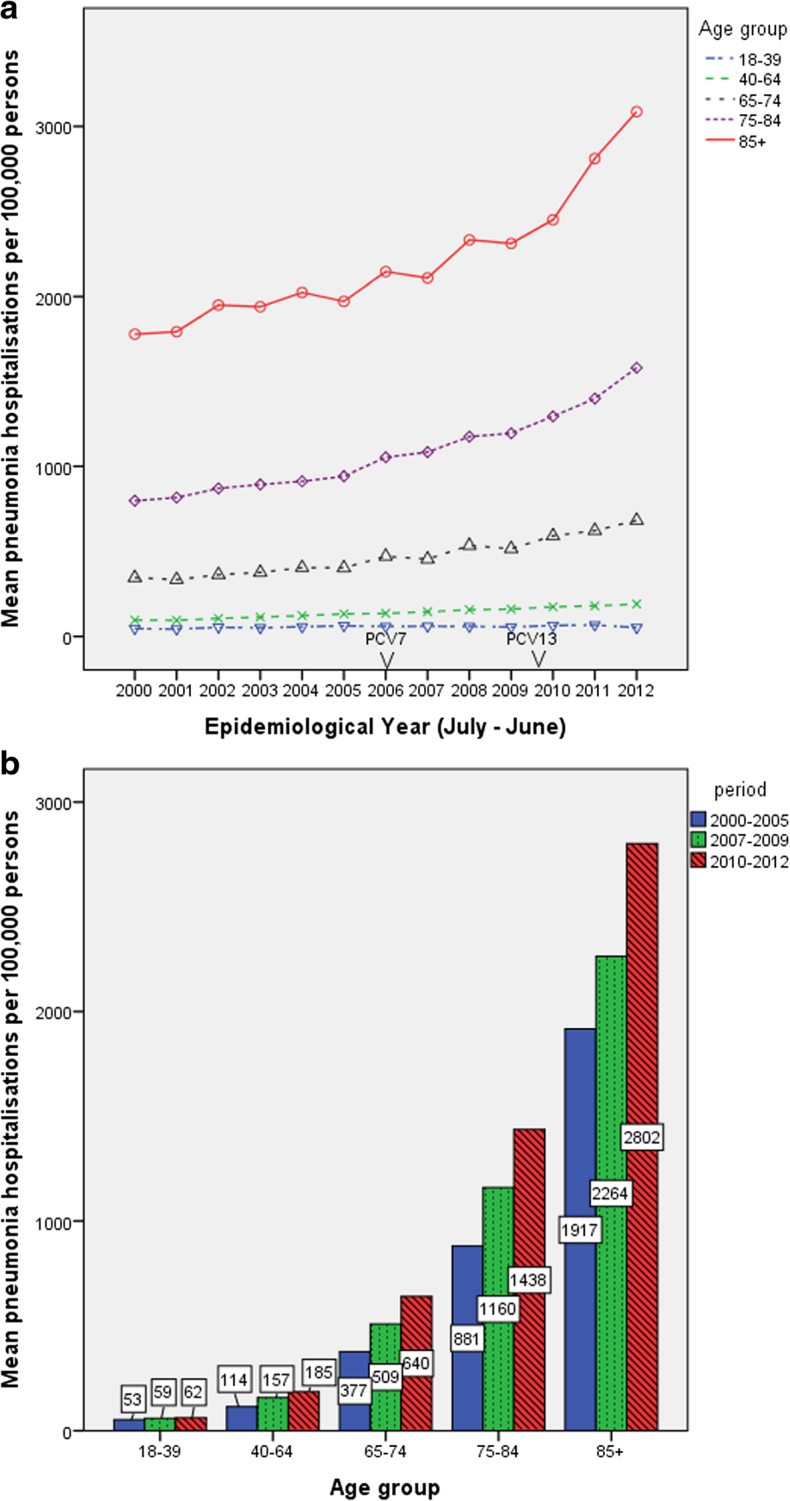
Pneumonia hospitalisations in Scotland from July 2000 to June 2012 in adults. Panel **a** shows annual rates of hospitalization for pneumonia from 2000 to 2012. PCV 7 was introduced in 2006 and PCV 13 in 2010. Panel **b** shows annual rates of hospitalization for 2000 to 2005, 2007 to 2009 and 2010 to 2012 for all adults (including those coded as “other” health boards)

From 2000 to 2012, there were 153,636 new hospitalisation episodes for pneumonia across Scotland. In addition to these hospitalisations, there were 4551 readmissions within 14 days of discharge. Pneumonia was the first listed diagnosis for 97.6 % of these hospitalisations. Sepsis was the first listed diagnosis for 1.9 %, and meningitis and empyema the first listed diagnosis each for 0.3 and 0.2 % respectively. Prior to the introduction of PCV, we observed a gradual increase in the rates of pneumonia hospitalisation in children aged below 5 years (Fig. 1). A similar pattern was seen in adults aged over 75 years (Fig. 2). Across the twelve year period (2000–2012), the annual rates of pneumonia hospitalisations were highest in adults aged above 65 years and children aged below two years (Table 1). In the pre-PCV period, the rates for pneumonia hospitalisation in adults aged 75 to 84 years and 85 years and above, were approximately 3 and 6.5 fold higher respectively than those in children aged less than two years. In the post-PCV 7 period, this difference increased to about 5 and 9 fold respectively; and further increased to about 7 and 13 fold respectively in the post-PCV 13 period. This pattern was sustained even when data from “other” Health Boards were excluded (Additional file 1: Figures S1 and S2).

**Table 1:** Rates of hospitalisation, length of hospital stay, and rates of in-hospital death related to pneumonia in Scotland 5 years before introduction of PCV 7 and three years after introduction of PCV 13

Age (years)	<2	2–4	5–17	18–39	40–64	65–74	75–84	85+
Pneumonia Hospitalisation rate /100K^a^ (95 % CI)								
2000–2005	293 (280–306)	181 (173–190)	43 (41–45)	53 (51–54)	112 (110–114)	373 (365–380)	875 (861–889)	1909 (1872–1946)
2010–2012	206 (191–221)	177 (166–188)	45 (42–48)	62 (59–64)	182 (179–186)	634 (621–647)	1426 (1402–1451)	2785 (2728–2842)
Lengths of hospital stay (days). Median (IQR)								
2000–2005	3 (1–5)	2 (1–4)	3 (1–5)	4 (2–6)	5 (3–10)	7 (4–14)	9 (4–19)	11 (5–25)
2010–2012	2 (1–5)	2 (1–4)	2 (1–5)	3 (1–6)	5 (2–9)	6 (3–12)	7 (4–15)	9 (4–19)
Lengths of hospital stay (days) incl. readmissions within 14 days. Median, IQR								
2000–2005	3 (1–5)	2 (1–4)	3 (1–5)	4 (2–7)	5 (3–11)	7 (4–15)	9 (4–20)	12 (4–25)
2010–2012	2 (1–5)	2 (1–4)	2 (1–5)	3 (1–7)	5 (2–9)	6 (3–12)	8 (4–16)	9 (4–19)
Reasons for admission coded as pneumococcal disease. %, 95 % CI								
2000–2005	9.3 (8.1–10.7)	3.3 (2.5–4.2)	4.1 (3.4–5.1)	6.8 (6.1–7.5)	6.1 (5.7–6.6)	3.8 (3.4–4.1)	2.3 (2.0–2.5)	1.1 (1.0–1.4)
2010–2012	3.3 (2.3, 4.9)	1.1 (0.6–1.9)	2.5 (1.7–3.6)	3.5 (2.9–4.3)	2.9 (2.6–3.3)	1.7 (1.4–2.0)	1.0 (0.8–1.2)	0.7 (0.5–0.9)
Deaths from pneumonia (primary cause, all patients)^b^ %, 95 % CI								
2000–2005	0	0.06 (0.008, 0.4)	0.2 (0.1, 0.6)	0.7 (0.5, 1.0)	3.5 (3.2, 3.8)	6.9 (6.5, 7.4)	13.1 (12.5, 13.6)	25.8 (24.9, 26.6)
2010–2012	0	0	0.3 (0.1, 0.9)	0.3 (0.1, 0.6)	1.4 (1.2, 1.7)	3.4 (3.0, 3.7)	6.2 (5.8, 6.6)	15.7 (15.0, 16.5)
Deaths from pneumonia as proportion of all deaths (primary cause)^c^ %, 95 % CI								
2000–2005	0	2.4 (0.3, 15.1)	3.1 (1.3, 7.4)	5.2 (3.7, 7.2)	7.4 (6.7, 8.2)	8.7 (8.1, 9.3)	14.1 (13.5, 14.7)	26.3 (25.4, 27.2)
2010–2012	0	0	11.1 (3.6, 29.3)	4.3 (2.2, 8.4)	6.0 (5.1, 7.1)	8.2 (7.4, 9.2)	12.0 (11.2, 12.8)	24.0 (22.9, 25.1)
Deaths from pneumonia (any cause, all patients)^d^ %, 95 % CI								
2000–2005	0.8 (0.5, 1.3)	0.6 (0.3, 1.0)	2.3 (1.8, 3.0)	3.7 (3.2, 4.3)	18.8 (18.1, 19.5)	36.9 (35.9, 37.8)	52.8 (52.0, 53.6)	66.1 (56.1, 67.0)
2010–2012	0.7 (0.3, 1.6)	0.2 (0.05, 0.8)	2.1 (1.4, 3.2)	2.4 (1.9, 3.1)	8.5 (8.0, 9.1)	14.9 (14.2, 15.6)	21.6 (20.9, 22.3)	36.1 (35.1, 37.1)

^a^The hospitalisation rates have been calculated after excluding data where patient’s health board was coded as “other”

^b^Deaths with pneumonia as a primary cause as a proportion of the whole population

^c^Deaths with pneumonia as a primary cause as a proportion of the deaths reported in this population

^d^Deaths with pneumonia as any cause as a proportion of the whole population

**Fig. 3:**
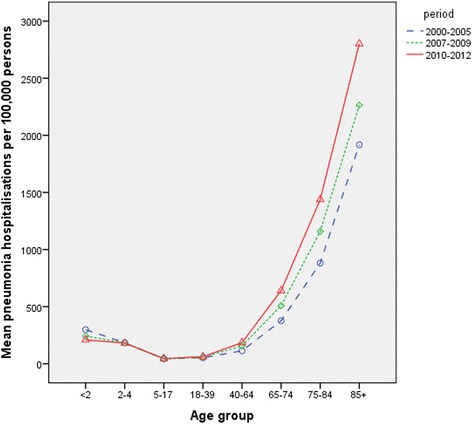
Average Annual Rates of Scottish hospitalisations for Pneumonia before and after the introduction of PCV7 and PCV13

The pneumonia hospitalisation rates in children aged less than 2 years decreased by about 30 % in the post-PCV 13 period compared to the pre-PCV period (Fig. 3). Pneumonia hospitalisations in this age group declined by about 19 % in the post-PCV7 period (compared to pre-PCV) and by about 14 % in the post-PCV13 period (compared to post-PCV7). However, the rates remained largely unchanged in the age groups 2–39 years. We saw that in the older age groups, the pneumonia hospitalisation rates increased steadily in the post-PCV period (greater increase post-PCV13 compared to post-PCV7). In adults aged 75–84 years and 85 years and above, the pneumonia hospitalisation rates increased by 63 and 46 % respectively in the post-PCV 13 period compared to the pre-PCV period (Table 1, Additional file 1: Table S2). This translates to an absolute increase of about 7000 hospitalisations across all age groups in Scotland in 2012. Half of this increase was observed in age groups older than 74 years (increase of 2200 and 1300 hospitalisations in the age group 75–84 years and 85 years and above respectively (Table 2).

**Table 2:** Difference in pneumonia hospitalisation rates in Scotland comparing pre-PCV7 and post-PCV13 periods. With absolute change in hospitalisations by 2012

Age group	Pneumonia hospitalisations in 2012	Scottish population 2012	Difference in hospitalisation rates per 100,000 population, PrePCV7 vs LatePCV7/PCV13 (n, 95 % CI)	Difference in hospitalisations, PrePCV7 to Late PCV7/PCV13 (%, 95 % CI)^a^	Expected number of HOSPITALISATIONS from 2000 to 2006 distribution^b^	Estimated absolute change in number of hospitalisations, 2012 (observed – expected), n, 95 % CI
<2	240	119,275	−87.0 (−107.0, −67.0)	−29.7 (−36.5, −22.8)	349.8	−109.8 (−125.7, −94.0)
2–4	283	176,596	−4.8 (−19.0, 9.0)	−2.8 (−10.5, 5.1)	320.9	−37.9 (−52.7, −23.0)
5–17	262	742,593	2.1 (−1.0, 5.4)	4.9 (−2.3, 12.6)	317.8	−55.8 (−69.9, −42.5)
18–39	783	1,506,987	9.1 (6.4, 11.9)	17.4 (12.2, 22.7)	789.7	−6.7 (−29.3, 15.9)
40–64	3521	1,842,398	70.3 (66.2, 74.4)	62.9 (59.2, 66.6)	2058.0	1463.0 (1424.4, 1500.0)
65–74	3467	507,265	262.0 (247.2, 276.7)	70.4 (66.4, 74.3)	1888.5	1578.5 (1541.4, 1615.5)
75–84	4888	309,244	551.6 (523.4, 579.7)	63.0 (59.8, 66.3)	2705.6	2182.4 (2138.8, 2226.0)
85+	3372	109,242	876.4 (808.7, 944.1)	45.9 (42.4, 49.5)	2085.4	1286.6 (1246.2, 1327.0)
All ages	16,816	5,313,600	110.3 (107.2, 113.3)	59.6 (57.9, 61.2)	9835.5	6980.5 (6895.5, 7060.2)

^a^As percentage of 2000–2006 (PrePCV7) data

^b^Derived from hospitalisation rate from PrePCV7 data applied to the 2012 population figures

We observed that during 2000–2012, the mean and median length of stay (LOS) increased with age (Additional file 1: Figure S3 and Additional file 1: Table S3). The pattern of longer stays (defined as more than 30 days) is the familiar “hook” pattern with the lowest number being in the 2–4 year age group and then increasing with age (Additional file 1: Table S4). This pattern is sustained even if a lower threshold (of stays more than 14 days) is used to define longer hospitalisation. In the post-PCV 13 period, median length of hospital stay decreased by a third in children aged below 2 years and by 22 and 18 % in the age groups 75–84 years and 85 years and above respectively compared with the pre-PCV period (Table 1). We also observed that although the distribution of length of stay in hospital (across all age groups) remained the same in the pre-PCV and post-PCV13 period, there was a significantly higher proportion of patients with stays longer than 14 days in the former (24.8 % vs 20.9 %, *p* < 0.001) (Additional file 1: Table S5). When the analysis was stratified by age, the pattern of significantly higher proportion of patients with stays longer than 14 days was seen only in adults aged over 40 years (Additional file 1: Table S5).

The proportion of pneumonia hospitalisations coded as pneumococcal disease decreased substantially across all age groups in the post-PCV13 period (Table 1). The most dramatic reduction was seen in children aged below 5 years (about a two-thirds reduction) while in the elderly aged 85 years only a one-third reduction was demonstrated. At a population level, we observed 29 (95 % CI 22 to 35) percent reduction in pneumococcal admissions across all age groups in Scotland in the post-PCV13 period compared to pre-PCV period. However, this varied by age group -while there was a 70 % reduction in pneumococcal hospitalisations in children aged below 5 years in the post-PCV13 period compared to pre-PCV period, older age groups experienced a more modest reduction (21 and 25 % in those 65–74 years and 75 years and above respectively) (Additional file 1: Table S6).

We observed a substantial reduction in the proportion of deaths due to pneumonia (as a primary cause) across all age groups in the post-PCV13 period when compared to pre-PCV period. In elderly patients aged 75–84 and 85 years and above there was a decrease of about 53 and 40 % respectively (Table 1). This pattern was sustained when deaths from pneumonia were defined more broadly and included all deaths in which pneumonia was mentioned on the death certificate but was not recorded as the underlying cause of death (Additional file 1: Table S7). At a population level, there was an overall 11 % reduction in deaths in post-PCV13 period compared to pre-PCV period, with a 19 and 12 % reduction in those aged 65–74 years and 75 years and above respectively (Additional file 1: Table S8). We also observed a 7 % reduction in pneumococcal deaths across all age groups with an 18 and 12 % reduction in the 65–74 years and 75 years and above age groups respectively (Additional file 1: Table S9).

## Discussion

This is the first national study to report the impact of a child PCV immunisation programme on pneumonia hospitalisations across all age groups. Our results are based on a Scottish national dataset for a 12 year period (2000–2012). We observed modest and sustained declines in the childhood hospitalisation rates for pneumonia in the six years following the introduction of PCV 7 and 13. This should alleviate concerns regarding pneumococcal serotype replacement, as we have found no evidence of increased pneumonia burden in children following the introduction of PCV. We also observed a 33 % reduction in the median length of stay in hospital for pneumonia in children aged below 2 years. However, we noted a dramatic and progressive increase in pneumonia hospitalisations in adults, most notably in those aged above 65 years. This translates to an annual increase of about 7000 hospital admissions for pneumonia across Scotland. This increase in hospital admissions is accompanied by contrasting decreases in the length of stay in hospital, the proportion of hospital admissions coded as pneumococcal pneumonia and pneumonia mortality in adults aged over 75 years.

We found a gradual increase in the annual pneumonia hospitalisation rates for children aged below 5 years in the pre-PCV period (Fig. 1). These results are consistent with those reported in previous analyses of pneumonia trends in Scotland and England prior to the introduction of PCV [16, 17]. Koshy and colleagues concluded that this was likely due to a true increase in disease incidence (reflected in hospitalisation rates), as it was consistent with similar reports elsewhere.

We reported a 30 % decline in pneumonia hospitalisations in children aged below 2 years following the introduction of first PCV 7 and later PCV 13, which is reassuringly consistent with results of clinical trials investigating the effectiveness of PCV against radiographically confirmed WHO Primary Endpoint Pneumonia (bacterial) pneumonia [18, 19]. A previous study from England reported 19 to 22 % decline in hospitalisation rates for bacterial pneumonia in children aged below 15 years in the two years post-PCV7 (2007 and 2008) [17]. Our results for the six years post-PCV in children aged below 18 years are consistent with those reported by Griffin and colleagues for US populations in the first decade after introduction of PCV 7 [14] (Panel 1).

However, in older adults, our results contrast markedly from findings reported by Griffin and colleagues [14]. We estimated that in Scotland, the hospitalisation rate for all-cause pneumonia in adults aged 65–74 years in the post-PCV13 period was 640.4 per 100,000 persons per year compared to 376.6 per 100,000 persons per year in the pre-PCV period. Similar increases were seen in the age groups 75 years and above. By contrast, Griffin and colleagues reported a slight decrease in the rate of hospitalisation in the post-PCV7 period (1208 compared to 1293 per 100,000 persons per year) in the 65–74 year age group. More substantial decreases were reported in the older age groups (75 years and above). In Scotland, this translates to an additional 7000 hospital admissions for pneumonia in post-PCV period, compared to 112,000 fewer hospitalisations in U.S adults. Griffin and colleagues attributed these declines to the indirect “herd immunity” effect of PCV on pneumococcal pneumonia. We did however observe a substantial decline in the proportion of pneumonia hospitalisations coded as pneumococcal disease across all age groups in the post-PCV period. However, it is very likely that we may have underestimated the impact of PCV on pneumococcal pneumonia. This is because hospital discharge data are based on clinical diagnoses recorded in patients’ medical records and accurate diagnosis of pneumococcal pneumonia is difficult without isolation of S. pneumoniae [20]. Notably, since 2010, we observed an increase in both the hospitalisations for pneumococcal pneumonia (numerator) and all-cause pneumonia (denominator) in adults older than 65 years even though there was a decrease in the proportion of pneumonia cases coded as pneumococcal pneumonia (Additional file 1: Table S11). Extrapolating from the pneumonia hospitalisation rates in the pre-PCV period, we calculated the (expected) number of pneumonia hospitalisations assuming absence of PCV immunisation in the period 2007–12. Unlike in younger children, the expected number of pneumonia hospitalisations during this period would have been lower in older children (aged above 5 years) and adults. Therefore, it appears that the impact of child PCV immunization in Scotland on pneumonia hospitalisations has been limited to children aged younger than 5 years (Table 2).

There are several reasons why our results are not consistent with those observed by Griffin and colleagues. Firstly, this could be attributed to differences in coding practices in both countries. The US Nationwide Inpatient Sample (NIS) database uses up to 15 diagnostic positions, whereas ISD only uses up to 6 diagnostic positions. Therefore for a first diagnosis of meningitis, septicaemia or empyema, pneumonia could have been a secondary diagnosis in one of 14 diagnostic positions in the US study, but a secondary diagnosis in only one of 5 diagnostic positions in the present study. This could mean that fewer cases of septicaemia, meningitis and empyema were included as pneumonia cases in Scotland. It has been reported that during 2003–09 in the US there was an observed decrease in the coding for a primary diagnosis of pneumonia, which was partially compensated for by increases in coding for primary diagnoses of other diseases e.g., sepsis and respiratory failure [21]. Therefore, the decline in hospitalisations for pneumonia reported in the US may be partially due to an artefact of changes in diagnostic coding. In Scotland, the percentage of hospitalisations with pneumonia as a secondary diagnosis has remained almost static at 2.5 % in the 2000–2005 period, 2.4 % in the 2007–2009 period and 2.5 % in the 2010–2012 period. Although the study by Griffin and colleagues would have included cases with a primary diagnosis of sepsis as pneumonia hospitalisation, they would not have included diagnoses that were coded as respiratory failure [14]. Indeed, Lindenauer and colleagues have reported that when combined with diagnoses of sepsis and respiratory failure, much more modest declines were seen in the hospitalisation rates for pneumonia [21]. Secondly, the US study was based on a 20 % sample of US hospitals, whereas our study was based on inpatient data from the whole of Scotland. Thirdly, the increasing trend of adult pneumonia hospitalisations in Scotland may be an artefact of changing healthcare practices. This could have overcompensated for any declines in adult all-cause pneumonia hospitalisations that may have been seen as a result of the known herd effect of PCV [13]. Indeed, there was a 10 % increase in all-cause hospitalisations (defined using CIS) during our study period (2000–2012) (data source: Information Services Division, National Services Scotland in response to data request IR2015-01696). Fourthly, the increase in pneumonia hospitalisations could be attributed to improvement in coding practices (even though ICD-10 codes have been in use in Scotland since 1996). Coding is also reported to have improved in England [22] and may have contributed to the apparent increases in pneumonia hospitalisations reported there [17]. In England and Wales, in the period 2000–10, there was an improvement in case ascertainment for invasive bacterial infections which is corroborated by a concomitant increase in cases of other invasive bacterial infections reported to Public Health England. Miller and colleagues corrected for these changes by retrospectively inflating the case count to the projected 2009–10 level of ascertainment [9]. They found that without adjusting for these changes there was little reported change in IPD incidence (across all age groups except children younger than 5 years) after PCV introduction although PCV has been demonstrated to have a substantial impact on IPD incidence both in children and adults [23].

The National Audit Office (NAO) has reported an increasing trend of emergency hospital admissions in Scotland and the rest of the UK [24]. The increase was thought to be partially due to an ageing population and due to a tendency to treat patients as day cases or give patients earlier discharge to reduce bed pressure, resulting in more readmissions. This is consistent with our observation of a trend towards decreasing length of hospital stay for patients with pneumonia. In England there was a 40 % increase in pneumonia hospitalisations for children aged less than 15 years between 1999 and 2010 [25]. Moreover, another study from England has reported a 34 % increase in hospital admissions for pneumonia before the introduction of PCV, mainly for pneumonias coded with an unspecified diagnosis [4]. Subsequent to changes to the general medical services contracts in 2004, General Practitioners in the UK (including Scotland) were no longer required to provide out of hours services. In England and Wales, this has resulted in a 32 % increase in A&E attendances between 2003–04 and 2013–14, translating into an 8 % growth in the likelihood of being admitted to hospital after attending A&E during this period [26]. Indeed, patients may be more likely to be sent to hospital by an unfamiliar doctor [4]. This is likely to be true for Scotland as well. With increased emergency department visits and referrals there would be a greater diagnosis of pneumonias that would have otherwise gone undiagnosed. We also observed an overall increase in the percentage of hospital readmissions for pneumonia from 2.0 % in 2000 to 3.6 % in 2012. We therefore considered episodes generated from readmissions within 14 days to be part of the same event and consequently excluded them from our primary analysis. However, when disaggregated by age groups, we observed that the proportion of early readmissions (within 14 days of discharge) decreased in those younger than 40 years (the most substantial decrease of about 74 % being in children aged below 2 years) (Additional file 1: Table S9). The US study also found a trend of decreasing hospital stays before and after the introduction of PCV and found decreases in pneumonia hospitalisation rates, even without adjusting for readmissions [14].

The difference between our findings and those reported by Griffin cannot be attributed to differences in pneumococcal polysaccharide vaccine (PPV) coverage in the two populations as they are likely to be similar for Scotland and U.S. Limited PPV coverage data for Scotland are available − the initial vaccination uptake was estimated to be 65 % [27]. Estimates for England indicate a coverage of about 69 % [28]. Similarly, PPV was introduced in the US in 1997 for all individuals aged 65 years and above. Although recent coverage data are not available, the data for 2005 estimate PPV coverage to be about 64 % [29].

## Conclusions

Our findings support the evidence on the impact of PCV on pneumonia hospitalisations in children, indicating that vaccine impact in this national programme is broadly consistent with predictions from vaccine trials. It is reassuring that reductions in hospitalization have persisted over 6 years with no evidence of negative indirect effects (due to serotype replacement) eroding this impact over this time period. This reduced proportion of pneumonia hospitalisations in children coded as pneumococcal disease post PCV13 introduction gives further reassurance that there is not a substantial serotype replacement effect over the study period. The failure to note any positive indirect effect of infant PCV vaccination on (pneumococcal) pneumonia in non-vaccinated individuals observed through measurement of hospitalisations among older individuals is likely due to contemporaneous changes in clinical practice and coding as discussed above. This suggests that indirect effects of PCV on observed hospitalisations may be context specific and demonstrates that health care planners and health economists cannot, with confidence, predict positive indirect PCV vaccine impacts on hospitalisations in their estimates of vaccine impact on health service costs. IPD surveillance in all age groups is needed to assess the indirect effects of PCV in the community.

## Abbreviations

CAP, community-acquired pneumonia; CHI, Community Health Index; CI, confidence interval; CIS, continuous inpatient stay; ICD, International classification of diseases; IPD, invasive pneumococcal disease; ISD, Information Services Division; LOS, length of stay; NAO, National Audit Office; NHS, National Health Service; NIS, Nationwide Inpatient Sample; NVT, non-vaccine serotypes; PCV, pneumococcal conjugate vaccine; PPV, pneumococcal polysaccharide vaccine; SHLMPRL, Scottish Haemophilus, Legionella, Meningococcus and Pneumococcus Reference Laboratory; UK, United Kingdom; US, United States

## Additional file

Additional file 1: Pneumonia hospitalisations in Scotland following the introduction of pneumococcal conjugate vaccination in young children. (DOCX 114 kb)
