# Supplementary material for: Pneumonia hospitalisations in Scotland following the introduction of pneumococcal conjugate vaccination in young children
Source: BMC Infect Dis. 2016 Aug 9;16:390. doi: 10.1186/s12879-016-1693-x (PMC4977871; doi:10.1186/s12879-016-1693-x)
Supplement: Additional file 1: — Pneumonia hospitalisations in Scotland following the introduction of pneumococcal conjugate vaccination in young children. (DOCX 114 kb) [file 12879_2016_1693_MOESM1_ESM.docx]

Supplementary material for

“Pneumonia hospitalisations in Scotland following the introduction of pneumococcal conjugate vaccination in young children”

Supplementary table 1: List of ICD-10 codes used for diagnosis

|  | ICD10 codes | ICD10 text | Comments |
| --- | --- | --- | --- |
| All cause pneumonia | J12.- | Viral pneumonia, not elsewhere classified |  |
|  | J13.- | Pneumonia due to Streptococcus pneumoniae |  |
|  | J14.- | Pneumonia due to Haemophilus influenzae |  |
|  | J15.- | Bacterial pneumonia, not elsewhere classified |  |
|  | J16.- | Pneumonia due to other infectious organisms, not elsewhere classified |  |
|  | J17.-^[[1]](#footnote-1)^* | Pneumonia in bacterial diseases classified elsewhere | As this is an 'asterisk code', J17.- would always be found in the second position, prior to 01/04/13, when it could be in either 1st or 2nd diagnostic position |
|  | J18.- | Pneumonia, organism unspecified |  |
|  |  |  |  |
|  | J10.0 | Influenza with pneumonia, influenza virus identified |  |
|  | J11.0 | Influenza with pneumonia, virus not identified |  |
|  |  |  |  |
|  | J85.1 | Abcess of lung with pneumonia | Excludes with J09 - J16 |
|  |  |  |  |
| All types/conditions of meningitis | G00 | bacterial meningitis, not elsewhere classified |  |
|  | G01* | Meningitis in bacterial diseases classified elsewhere | As this is an 'asterisk code', G01 would always be found in the second position prior to 01/04/13, when it could be in either 1st or 2nd |
|  | G02* | Meningitis in other infectious and parasitic diseases classified elsewhere | As this is an 'asterisk code', G02 would always be found in the second position prior to 01/04/13, when it could be in either 1st or 2nd |
|  | G03 | Meningitis due to other and unspecified causes |  |
|  | A87- | Viral meningitis, unspecified |  |
|  | G00-G05, as well as A83.5, A84.1 and A86. |  |  |
|  | B45.1 | cryptococcal meningitis |  |
|  | A17.0 | Tuberculous meningitis |  |
|  | A42.81 | Actinomycotic meningitis |  |
|  | B37.5 | Candidal meningitis |  |
|  | A17.0 | Caseous (tuberculous) |  |
|  | A02.21 | Salmonella meningitis |  |
|  | A39.0 | Meningococcal menigitis |  |
|  | B02.1 | Herpes zoster meningitis |  |
|  | B00.3 | herpes simplex meningitis |  |
|  | B26.1 | Mumps meningitis |  |
|  | A52.13 | syphillitic meningitis |  |
|  | A54.81 | Gonococcal |  |
|  | A27.81 | leptospiral meningitis |  |
|  | B38.4 | Coccidiodomycosis meningitis |  |
|  |  |  |  |
| All types/conditions of septicemia |  |  |  |
|  | A02.1 | Salmonella sepsis |  |
|  | A20.7 | Septicaemia plague |  |
|  | A22.7 | Anthrax sepsis |  |
|  | A39.2 | Acute meningococcaemia |  |
|  | A39.3 | Chronic meningococcaemia |  |
|  | A39.4 | Meningococcaemia, unspecified | Neutropenic - + D60.X |
|  | A40.- | Streptococcal sepsis |  |
|  | A41.- | Other sepsis | Title was "Other septicaemia" in Scotland prior to 01/04/13. |
|  | R57.2 | Septic shock | New code in V4 |
|  | R65.1 | Systemic Inflammatory Response syndrome of infectious origin with organ failure | New code in V4 0 includes severe sepsis |
|  | B00.7 | Herpes viral sepsis |  |
|  | R65.21 | Septic shock |  |
|  | R78.81 | bacteremia |  |
|  | R65.20 | Sever sepsis w/o shock |  |
|  | J86 | Pyothorax includes empyema |  |
|  |  |  |  |
|  |  |  |  |
| All pneumococcal pneumonia codes | J13.X | Pneumonia due to Streptococcus pneumoniae |  |
|  | (A40.3) | Sepsis due to s. pneumoniae |  |
|  | (G00.1) | Pneumococcal meningitis |  |
|  | B95.3 | S. Pneumoniae as cause of diseases classified elsewhere |  |

Supplementary table 2: Expected number of pneumonia hospitalisations in Scotland in the post-PCV13 period (2010-2012) extrapolating the rates during pre-PCV period (2000-2005)

| Age group (years) | Pneumonia hospitalisation rate (per 100,000 persons per year) in 2000-2005 | Observed number of pneumonia hospitalisations in 2010-2012 | Scottish Population  2010-2012 | Expected number of pneumonia hospitalisations from 2000-2005 rate | χ^2^ contribution^[[2]](#footnote-2)^ |
| --- | --- | --- | --- | --- | --- |
| <2 | 298.65 | 748 | 356691 | 1065 | 94.49 |
| 2-4 | 183.85 | 951 | 526286 | 968 | 0.28 |
| 5-17 | 43.55 | 1015 | 2235384 | 973 | 1.77 |
| 18-39 | 53.25 | 2817 | 4513767 | 2403 | 71.11 |
| 40-64 | 113.62 | 10161 | 5501865 | 6251 | 2445.34 |
| 65-74 | 376.56 | 9388 | 1465902 | 5520 | 2710.40 |
| 75-84 | 881.49 | 13126 | 912815 | 8046 | 3206.74 |
| 85+ | 1916.94 | 9047 | 322890 | 6190 | 1319.10 |

Supplementary table 3: Distribution of length of stay in hospital by age band

| Length of stay (days) | | | | | | |
| --- | --- | --- | --- | --- | --- | --- |
| Age Band (years) | Mean | N | Std. Deviation | Median | Minimum | Maximum |
| <2 | 4.7128 | 3962 | 12.51797 | 2.0000 | .00 | 378.00 |
| 2-4 | 3.5320 | 4162 | 11.74038 | 2.0000 | .00 | 620.00 |
| 5-17 | 4.3432 | 4729 | 9.41030 | 2.0000 | .00 | 368.00 |
| 18-39 | 6.3878 | 11604 | 24.06791 | 3.0000 | .00 | 1564.00 |
| 40-64 | 9.3346 | 33252 | 23.54174 | 5.0000 | .00 | 2463.00 |
| 65-74 | 12.0221 | 29652 | 27.76710 | 7.0000 | .00 | 2974.00 |
| 75-84 | 14.9796 | 42171 | 25.24244 | 8.0000 | .00 | 1859.00 |
| 85+ | 18.1285 | 28655 | 28.68479 | 10.0000 | .00 | 1096.00 |
| Total | 12.3024 | 158187 | 25.50422 | 6.0000 | .00 | 2974.00 |

Supplementary table 4: Distribution of duration of hospitalisation (grouped) by age band

|  | | | Length of stay | | | | | | Total |
| --- | --- | --- | --- | --- | --- | --- | --- | --- | --- |
|  |  |  | 0-1 days | 2-4 days | 5-7 days | 8-14 days | 15-30 days | 31+ |  |
| Age band (years) | <2 | Count | 1260 | 1677 | 494 | 322 | 161 | 48 | 3962 |
|  |  | % within Age band | 31.8% | 42.3% | 12.5% | 8.1% | 4.1% | 1.2% | 100.0% |
|  | 2-4 | Count | 1564 | 1840 | 374 | 263 | 95 | 26 | 4162 |
|  |  | % within Age band | 37.6% | 44.2% | 9.0% | 6.3% | 2.3% | .6% | 100.0% |
|  | 5-17 | Count | 1544 | 1997 | 535 | 413 | 189 | 51 | 4729 |
|  |  | % within Age band | 32.6% | 42.2% | 11.3% | 8.7% | 4.0% | 1.1% | 100.0% |
|  | 18-39 | Count | 2798 | 4394 | 2085 | 1414 | 612 | 301 | 11604 |
|  |  | % within Age band | 24.1% | 37.9% | 18.0% | 12.2% | 5.3% | 2.6% | 100.0% |
|  | 40-64 | Count | 5539 | 9581 | 6869 | 6186 | 3352 | 1725 | 33252 |
|  |  | % within Age band | 16.7% | 28.8% | 20.7% | 18.6% | 10.1% | 5.2% | 100.0% |
|  | 65-74 | Count | 3543 | 6793 | 5981 | 6806 | 4229 | 2300 | 29652 |
|  |  | % within Age band | 11.9% | 22.9% | 20.2% | 23.0% | 14.3% | 7.8% | 100.0% |
|  | 75-84 | Count | 4287 | 7841 | 7368 | 10088 | 7576 | 5011 | 42171 |
|  |  | % within Age band | 10.2% | 18.6% | 17.5% | 23.9% | 18.0% | 11.9% | 100.0% |
|  | 85+ | Count | 2836 | 4631 | 4124 | 6422 | 6008 | 4634 | 28655 |
|  |  | % within Age band | 9.9% | 16.2% | 14.4% | 22.4% | 21.0% | 16.2% | 100.0% |
| Total | | Count | 23371 | 38754 | 27830 | 31914 | 22222 | 14096 | 158187 |
|  |  | % within Age band | 14.8% | 24.5% | 17.6% | 20.2% | 14.0% | 8.9% | 100.0% |

Supplementary table 5: Comparison of duration of hospitalisation for pneumonia stratified by age in the pre-PCV and post-PCV13 period

| Age band (years) | | | period | | Total |
| --- | --- | --- | --- | --- | --- |
|  |  |  | 2000-2005 | 2010-2012 |  |
| <2 | Length of stay | 0 (day case) | 154 (8.1%) | 116 (15.5%) | 270 |
|  |  | 1 (overnight) | 366 (19.3%) | 146 (19.5%) | 512 |
|  |  | 2-3 days | 684 (36.1%) | 216 (28.9%) | 900 |
|  |  | 4-5 days | 301 (15.9%) | 100 (13.4%) | 401 |
|  |  | 6-10 days | 225 (11.9%) | 97 (13.0%) | 322 |
|  |  | 11-14 days | 66 (3.5%) | 29 (3.9%) | 95 |
|  |  | >14 days | 99 (5.2%) | 44 (5.9%) | 143 |
|  | Total | | 1895 | 748 | 2643 |
| 2-4 | Length of stay | 0 (day case) | 212 (11.7%) | 161 (16.9%) | 373 |
|  |  | 1 (overnight) | 406 (22.4%) | 213 (22.4%) | 619 |
|  |  | 2-3 days | 734 (40.5%) | 333 (35.0%) | 1067 |
|  |  | 4-5 days | 212 (11.7%) | 99 (10.4%) | 311 |
|  |  | 6-10 days | 158 (8.7%) | 94 (9.9%) | 252 |
|  |  | 11-14 days | 41 (2.3%) | 16 (1.7%) | 57 |
|  |  | >14 days | 49 (2.7%) | 35 (3.7%) | 84 |
|  | Total | | 1812 | 951 | 2763 |
| 5-17 | Length of stay | 0 (day case) | 244 (11.5%) | 154 (15.2%) | 398 |
|  |  | 1 (overnight) | 378 (17.8%) | 189 (18.6%) | 567 |
|  |  | 2-3 days | 762 (35.9%) | 328 (32.3%) | 1090 |
|  |  | 4-5 days | 319 (15.0%) | 149 (14.7%) | 468 |
|  |  | 6-10 days | 234 (11.0%) | 98 (9.7%) | 332 |
|  |  | 11-14 days | 79 (3.7%) | 42 (4.1%) | 121 |
|  |  | >14 days | 105 (5.0%) | 55 (5.4%) | 160 |
|  | Total | | 2121 | 1015 | 3136 |
| 18-39 | Length of stay | 0 (day case) | 348 (7.2%) | 304 (10.8%) | 652 |
|  |  | 1 (overnight) | 645 (13.3%) | 453 (16.1%) | 1098 |
|  |  | 2-3 days | 138 (28.5%) | 764 (27.1%) | 2145 |
|  |  | 4-5 days | 975 (20.1%) | 467 (16.6%) | 1442 |
|  |  | 6-10 days | 887 (18.3%) | 493 (17.5%) | 1380 |
|  |  | 11-14 days | 232 (4.8%) | 124 (4.4%) | 356 |
|  |  | >14 days | 371 (7.7%) | 212 (7.5%) | 583 |
|  | Total | | 4839 | 2817 | 7656 |
| 40-64 | Length of stay | 0 (day case) | 664 (5.7%) | 669 (6.6%) | 1333 |
|  |  | 1 (overnight) | 953 (8.4%) | 1143 (11.2%) | 2096 |
|  |  | 2-3 days | 2040 (18.1%) | 2156 (21.2%) | 4196 |
|  |  | 4-5 days | 2079 (18.4%) | 1822 (17.9%) | 3901 |
|  |  | 6-10 days | 2788 (24.7%) | 2268 (22.3%) | 5056 |
|  |  | 11-14 days | 897 (7.9%) | 733 (7.2%) | 1630 |
|  |  | >14 days | 1868 (16.5%) | 1370 (13.5%) | 3238 |
|  | Total | | 11289 | 10161 | 21450 |
| 65-74 | Length of stay | 0 (day case) | 454 (4.5%) | 413 (4.4%) | 867 |
|  |  | 1 (overnight) | 663 (6.5%) | 735 (7.8%) | 1398 |
|  |  | 2-3 days | 1279 (12.6%) | 1580 (16.8%) | 2859 |
|  |  | 4-5 days | 1470 (14.4%) | 1596 (17.0%) | 3066 |
|  |  | 6-10 days | 2722 (26.7%) | 2379 (25.3%) | 5101 |
|  |  | 11-14 days | 1108 (10.9%) | 872 (9.3%) | 1980 |
|  |  | >14 days | 2488 (24.4%) | 1813 (19.3%) | 4301 |
|  | Total | | 10184 | 9388 | 19572 |
| 75-84 | Length of stay | 0 (day case) | 514 (3.5%) | 436 (3.3%) | 950 |
|  |  | 1 (overnight) | 885 (6.0%) | 966 (7.4%) | 1851 |
|  |  | 2-3 days | 1534 (10.4%) | 1864 (14.2%) | 3398 |
|  |  | 4-5 days | 1661 (11.3%) | 1816 (13.8%) | 3477 |
|  |  | 6-10 days | 3494 (23.8%) | 3254 (24.8%) | 6748 |
|  |  | 11-14 days | 1660 (11.3%) | 1401 (10.7%) | 3061 |
|  |  | >14 days | 4951 (33.7%) | 3389 (25.8%) | 8340 |
|  | Total | | 14699 | 13126 | 27825 |
| 85+ | Length of stay | 0 (day case) | 318 (3.2%) | 308 (3.4%) | 626 |
|  |  | 1 (overnight) | 605 (6.0%) | 638 (7.1%) | 1243 |
|  |  | 2-3 days | 982 (9.7%) | 1120 (12.4%) | 2102 |
|  |  | 4-5 days | 913 (9.1%) | 986 (10.9%) | 1899 |
|  |  | 6-10 days | 1947 (19.3%) | 2051 (22.7%) | 3998 |
|  |  | 11-14 days | 1147 (11.4%) | 985 (10.9%) | 2132 |
|  |  | >14 days | 4176 (41.4%) | 2959 (32.7%) | 7135 |
|  | Total | | 10088 | 9047 | 19135 |
| Total | Length of stay | 0 (day case) | 2908 (5.1%) | 2561 (5.4%) | 5469 |
|  |  | 1 (overnight) | 4901 (8.6%) | 4483 (9.5%) | 9384 |
|  |  | 2-3 days | 9396 (16.5%) | 8361 (17.7%) | 17757 |
|  |  | 4-5 days | 7930 (13.9%) | 7035 (14.9%) | 14965 |
|  |  | 6-10 days | 12455 (21.9%) | 10734 (22.7%) | 23189 |
|  |  | 11-14 days | 5230 (9.2%) | 4202 (8.9%) | 9432 |
|  |  | >14 days | 14107 (24.8%) | 9877 (20.9%) | 23984 |
|  | Total | | 56927 | 47253 | 104180 |

Supplementary table 6: Hospital admissions for pneumococcal pneumonia in pre-PCV7 and post-PCV13 periods

| Age group | Pneumococcal admissions in 2010-2012 (post-PCV13) | Scottish Population 2010-2012 | Difference in death rates per 100,000 population, PrePCV7 vs PostPCV13 (n, 95% CI) | Difference in pneumococcal admissions, PrePCV7 to PostPCV13  (%, 95% CI)^[[3]](#footnote-3)^ | Expected number of pneumococcal admissions from 2000-2005 (PrePCV7) distribution^[[4]](#footnote-4)^ | Estimated Absolute Change in Number of pneumococcal admissions, 2010-2012 (observed – expected), n, 95% CI |
| --- | --- | --- | --- | --- | --- | --- |
| <5 | 34 | 882977 | -10.0 (-13.0, -8.0) | -69.9 (-90.9, -55.9) | 126.3 | -92.3 (-108.2, -75.5) |
| 5-17 | 25 | 2235384 | -0.6 (-1.0, -0.05) | -36.9 (-58.8, -3.1) | 38.0 | -13.0 (-21.9, -6.3) |
| 18-64 | 396 | 10015632 | -1.0 (-2.0, -0.8) | -18.9 (-37.7, -15.2) | 530.8 | -134.8 (-164.9, -94.8) |
| 65-74 | 155 | 1465902 | -3.0 (-6.0, -1.0) | -21.4 (-42.9, -7.1) | 205.2 | -50.2 (-70.7, -29.7) |
| 75+ | 189 | 1235705 | -5.0 (-8.0, -2.0) | -24.9 (-39.8, -10.0) | 248.4 | -59.4 (-82.9, -35.9) |
| All Ages | 799 | 15835600 | -2.0 (-2.5, -1.5) | -28.6 (-35.1, -22.0) | 1108.5 | -309.5 (-357, -262) |

Supplementary table 7: Pneumonia admissions, all-cause deaths, and pneumonia deaths (where pneumonia was primary cause) in hospital 2000-2012

|  | | | Deaths in patients admitted with pneumonia | Death from pneumonia (primary cause) | Pneumonia admissions |
| --- | --- | --- | --- | --- | --- |
| Epidemiological Year (July - June) | 2000 | Count | 6042 | 885 | 8619 |
|  |  | % within Epidemiological Year (July - June) | 70.1% | 10.3% |  |
|  | 2001 | Count | 6066 | 904 | 8716 |
|  |  | % within Epidemiological Year (July - June) | 69.6% | 10.4% |  |
|  | 2002 | Count | 6456 | 997 | 9566 |
|  |  | % within Epidemiological Year (July - June) | 67.5% | 10.4% |  |
|  | 2003 | Count | 6613 | 1044 | 9812 |
|  |  | % within Epidemiological Year (July - June) | 67.4% | 10.6% |  |
|  | 2004 | Count | 6745 | 982 | 10497 |
|  |  | % within Epidemiological Year (July - June) | 64.3% | 9.4% |  |
|  | 2005 | Count | 6763 | 961 | 11193 |
|  |  | % within Epidemiological Year (July - June) | 60.4% | 8.6% |  |
|  | 2006 | Count | 7399 | 1084 | 11933 |
|  |  | % within Epidemiological Year (July - June) | 62.0% | 9.1% |  |
|  | 2007 | Count | 7270 | 1081 | 12102 |
|  |  | % within Epidemiological Year (July - June) | 60.1% | 8.9% |  |
|  | 2008 | Count | 7687 | 1112 | 13330 |
|  |  | % within Epidemiological Year (July - June) | 57.7% | 8.3% |  |
|  | 2009 | Count | 7397 | 999 | 13511 |
|  |  | % within Epidemiological Year (July - June) | 54.7% | 7.4% |  |
|  | 2010 | Count | 7382 | 963 | 14892 |
|  |  | % within Epidemiological Year (July - June) | 49.6% | 6.5% |  |
|  | 2011 | Count | 6887 | 955 | 16369 |
|  |  | % within Epidemiological Year (July - June) | 42.1% | 5.8% |  |
|  | 2012 | Count | 5633 | 865 | 17647 |
|  |  | % within Epidemiological Year (July - June) | 31.9% | 4.9% |  |
| Total | | Count | 88340 | 12832 | 158187 |
|  |  | % within Epidemiological Year (July - June) | 55.8% | 8.1% |  |

Supplementary table 8: Pneumonia deaths in the pre-PCV7 and post-PCV13 periods

| Age group | Deaths in 2010-2012 (post-PCV13) | Scottish Population 2010-2012 | Difference in death rates per 100,000 population, PrePCV7 vs post-PCV13 (n, 95% CI) | Difference in deaths, PrePCV7 to PostPCV13  (%, 95% CI)^[[5]](#footnote-5)^ | Expected number of deaths from 2000-2005 (PrePCV7) distribution^[[6]](#footnote-6)^ | Estimated Absolute Change in Number of deaths, 2010-2012 (observed – expected), n, 95% CI |
| --- | --- | --- | --- | --- | --- | --- |
| <64 | 156 | 13133993 | -0.5 (-0.7, -0.3) | -29.5 (-43.9, -15.1) | 223.3 | -67.3 (-93.5, -41.0) |
| 65-74 | 312 | 1465902 | -5.0 (-8.0, -2.0) | -19.2 (-30.7, -7.7) | 382.6 | -70.6 (-98.5, -41.3) |
| 75+ | 2228 | 1235705 | -25.0 (-34.0, -15.0) | -12.2 (-16.6, -7.3) | 2535.7 | -307.7 (-381.8, -233.5) |
| All Ages | 2699 | 18953961 | -2.0 (-2.0, -0.7) | -10.8 (-10.8, -3.8) | 3506.5 | -807.5 (-902.3, -731.7) |

Supplementary table 9: Number of deaths due to pneumococcal pneumonia and mortality rate (per 100,000 population)

| Age group | Number of deaths in 2000-2005 (mortality rate per 100,000 population) | Number of deaths in 2007-2009 (mortality rate per 100,000 population) | Number of deaths in 2010-2010 (mortality rate per 100,000 population) |
| --- | --- | --- | --- |
| <5 | 1/1620092 (0.06) | 1/847161(0.1) | 0/882977 (0) |
| 5-17 | 5/4870656 (0.1) | 4/2288086 (0.2) | 3/2235384 (0.1) |
| 18-64 | 425/19023536 (2.2) | 229/9800785 (2.3) | 153/10015632 (1.5) |
| 65-74 | 705/2704480 (26.1) | 311/1390731 (22.4) | 312/1465902 (21.3) |
| 75+ | 4502/2193776 (205.2) | 2572/1179937 (218.0) | 2228/1235705 (180.3) |
| All Ages | 5644/36903288 (15.3) | 3122/18641947 (16.7) | 2699/18953961 (14.2) |

Supplementary table 10: Number and proportion (as %) of readmissions for pneumonia within 14 days between 2000 and 2012 by age bands

|  | | | period | | | Total |
| --- | --- | --- | --- | --- | --- | --- |
|  |  |  | 2000-2005 | 2007-2009 | 2010-2012 |  |
| Age band (years) | <2 | Count | 109 | 41 | 31 | 181 |
|  |  | % within period | 7.4% | 3.7% | 1.9% | 4.3% |
|  | 2-4 | Count | 82 | 27 | 46 | 155 |
|  |  | % within period | 5.6% | 2.5% | 2.8% | 3.7% |
|  | 5-17 | Count | 91 | 35 | 49 | 175 |
|  |  | % within period | 6.2% | 3.2% | 3.0% | 4.1% |
|  | 18-39 | Count | 157 | 102 | 124 | 383 |
|  |  | % within period | 10.6% | 9.3% | 7.5% | 9.1% |
|  | 40-64 | Count | 313 | 245 | 390 | 948 |
|  |  | % within period | 21.2% | 22.3% | 23.6% | 22.4% |
|  | 65-74 | Count | 221 | 214 | 329 | 764 |
|  |  | % within period | 15.0% | 19.5% | 19.9% | 18.1% |
|  | 75-84 | Count | 317 | 294 | 429 | 1040 |
|  |  | % within period | 21.5% | 26.8% | 25.9% | 24.6% |
|  | 85+ | Count | 186 | 139 | 257 | 582 |
|  |  | % within period | 12.6% | 12.7% | 15.5% | 13.8% |
| Total | | Count | 1476 | 1097 | 1655 | 4228 |
|  |  | % within period | 100.0% | 100.0% | 100.0% | 100.0% |

Supplementary table 11: Hospital admissions for pneumococcal pneumonia between 2000 and 2012 by age bands

| Age group | Period | Pneumococcal total | Pneumonia total | Pneumococcal pneumonia as a proportion of all pneumonia admissions (%) |
| --- | --- | --- | --- | --- |
| <2 | 2000-2005 | 177 | 1895 | 9.3 |
|  | 2007-2009 | 32 | 847 | 3.8 |
|  | 2010-2012 | 25 | 748 | 3.3 |
|  | Total | 234 | 3490 | 6.7 |
| 2-4 | 2000-2005 | 59 | 1812 | 3.3 |
|  | 2007-2009 | 13 | 911 | 1.4 |
|  | 2010-2012 | 10 | 951 | 1.1 |
|  | Total | 82 | 3674 | 2.2 |
| 5-17 | 2000-2005 | 88 | 2121 | 4.1 |
|  | 2007-2009 | 26 | 960 | 2.7 |
|  | 2010-2012 | 25 | 1015 | 2.5 |
|  | Total | 139 | 4096 | 3.4 |
| 18-39 | 2000-2005 | 328 | 4839 | 6.8 |
|  | 2007-2009 | 121 | 2638 | 4.6 |
|  | 2010-2012 | 100 | 2817 | 3.5 |
|  | Total | 549 | 10294 | 5.3 |
| 40-64 | 2000-2005 | 689 | 11289 | 6.1 |
|  | 2007-2009 | 352 | 8386 | 4.2 |
|  | 2010-2012 | 299 | 10161 | 2.9 |
|  | Total | 1340 | 29836 | 4.5 |
| 65-74 | 2000-2005 | 383 | 10184 | 3.8 |
|  | 2007-2009 | 138 | 7079 | 1.9 |
|  | 2010-2012 | 157 | 9388 | 1.7 |
|  | Total | 678 | 26651 | 2.5 |
| 75-84 | 2000-2005 | 332 | 14699 | 2.3 |
|  | 2007-2009 | 116 | 10181 | 1.1 |
|  | 2010-2012 | 127 | 13126 | 1.0 |
|  | Total | 575 | 38006 | 1.5 |
| 85+ | 2000-2005 | 115 | 10088 | 1.1 |
|  | 2007-2009 | 44 | 6844 | 0.6 |
|  | 2010-2012 | 63 | 9047 | 0.7 |
|  | Total | 222 | 25979 | 0.9 |
| Total | 2000-2005 | 2171 | 56927 | 3.8 |
|  | 2007-2009 | 842 | 37846 | 2.2 |
|  | 2010-2012 | 806 | 47253 | 1.7 |
|  | Total | 3819 | 142026 | 2.7 |

Supplementary table 12: Pneumonia admissions, all-cause deaths, and pneumonia deaths (where pneumonia was primary cause) in hospital 2000-2012


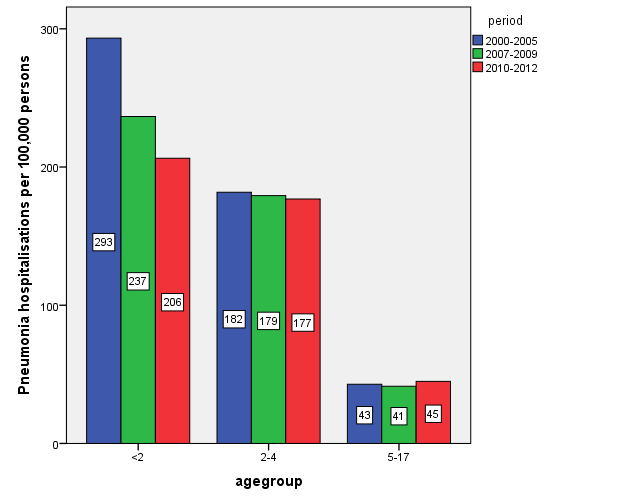


Supplementary figure 1: Annual rate of pneumonia hospitalisations in Scotland from July 2000 to July 2012 in children after excluding data from “other health boards”


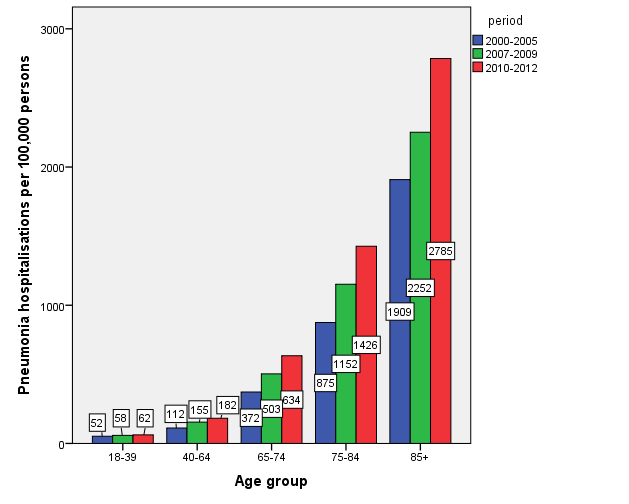


Supplementary figure 2: Annual rate of pneumonia hospitalisations in Scotland from July 2000 to July 2012 in adults after excluding data from “other health boards”


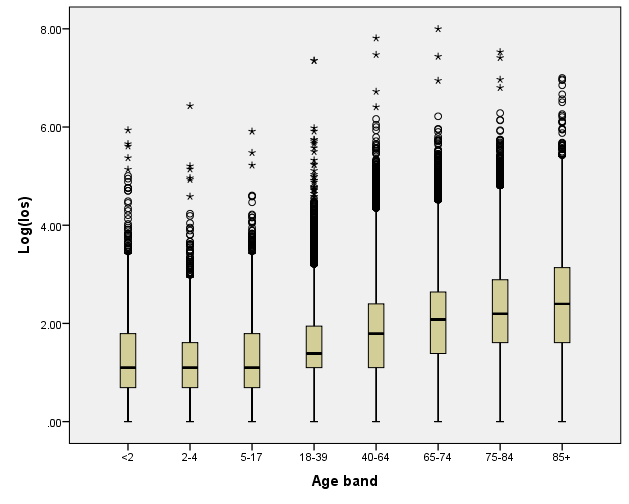


Supplementary figure 3: Length of stay in hospital in hospital (on logarithmic scale) by age group

1. Asterisk indicates that this code is located on 2nd diagnosis position only prior to 01/04/13 [↑](#footnote-ref-1)
2. As the χ^2^distribution threshold for 1 degree of freedom (i.e one age group) at p=0.01 (1%) is 6.63, all except 2-4 and 5-17 years are significant changes over time. If we look at age groups above 65 years, the total contribution is 19755.98, p<0.000001. [↑](#footnote-ref-2)
3. As percentage of 2000-2005 (PrePCV7) data [↑](#footnote-ref-3)
4. Hospitalisation rate from PrePCV7 data applied to the PostPCV13 (2012) population estimates [↑](#footnote-ref-4)
5. As percentage of 2000-2005 (PrePCV7) data [↑](#footnote-ref-5)
6. Hospitalisation rate from PrePCV7 data applied to the PostPCV13 (2012) population estimates [↑](#footnote-ref-6)
